# Bystanders intervene to impede grooming in Western chimpanzees and sooty mangabeys

**DOI:** 10.1098/rsos.171296

**Published:** 2017-11-08

**Authors:** Alexander Mielke, Liran Samuni, Anna Preis, Jan F. Gogarten, Catherine Crockford, Roman M. Wittig

**Affiliations:** 1 Department of Primatology, Max Planck Institute for Evolutionary Anthropology, Leipzig, Germany; 2 Taï Chimpanzee Project, Centre Suisse de Recherches Scientifiques en Côte d'Ivoire, Abidjan, Côte d'Ivoire; 3 Department of Biology, McGill University, Montreal, Canada; 4 P3: “Epidemiology of Highly Pathogenic Microorganisms”, Robert Koch Institute, Berlin, Germany

**Keywords:** chimpanzee, sooty mangabey, grooming, interventions, bystander

## Abstract

Grooming interactions benefit groomers, but may have negative consequences for bystanders. Grooming limits bystanders' grooming access and ensuing alliances could threaten the bystander's hierarchy rank or their previous investment in the groomers. To gain a competitive advantage, bystanders could intervene into a grooming bout to increase their own grooming access or to prevent the negative impact of others' grooming. We tested the impact of dominance rank and social relationships on grooming intervention likelihood and outcome in two sympatric primate species, Western chimpanzees (*Pan troglodytes verus*) and sooty mangabeys (*Cercocebus atys atys*). In both species, rather than increasing their own access to preferred partners, bystanders intervened mainly when an alliance between groomers could have a negative impact on them: when the lower-ranking groomer was close to the bystander in rank, when either groomer was an affiliation partner whose services they could lose, or the groomers were not yet strongly affiliated with each other. Thus, bystanders in both species appear to monitor grooming interactions and intervene based on their own dominance rank and social relationships, as well as triadic awareness of the relationship between groomers. While the motivation to intervene did not differ between species, mangabeys appeared to be more constrained by dominance rank than chimpanzees.

## 1. Introduction

Grooming and other affiliative behaviours are low-cost forms of cooperation that have been argued to play a vital role in shaping social relationships in many animal species [1]. They are thought to be used in exchange for grooming itself [2–4] and other services [5], and to establish alliances and maintain long-term cooperative exchanges in the form of social bonds [6–9]. Alliances and bonds impact individual fitness [10–12] by facilitating coalitionary support [5,13–18], which can lead to increased rank, which is positively correlated with fitness [16,17,19]. There is mounting evidence that individuals benefit by exchanging grooming for grooming and the resulting parasite removal and physiological benefits [20–22]; also, dyads that groom more show increased mating [23–25], food sharing [26–28], feeding tolerance [5,29,30], protection of infants [31,32] and access to infants [33–35]. Since the services an individual can offer in return for grooming differ and individuals have limited time to allocate grooming, competition for grooming partners arises [36–38].

In primates, individuals have been shown to bias their grooming towards higher-ranking grooming (HRG) partners [36,37,39–44], individuals close in rank [39,45–47], kin [37,48], bond partners [44], mothers with infants [33,34] and previous grooming partners [3,4,42,49]. The grooming distribution found in a social group is predicted to be the result of each group member attempting to maximize the benefit they receive from grooming [45], with some individuals having priority of access because of their higher rank and ability to monopolize resources, restricting others' access [36]. Previous studies have recognized that beyond priority of access, bystander behaviour can influence grooming bouts [43,45,50,51], but quantifying the importance of bystanders has proved difficult given the continued use of correlational measures to study grooming in primates [3,45,52].

The triadic nature of competitive events is lost when aggregating the distribution of multiple grooming bouts over time, impeding our understanding of grooming competition. While grooming has mainly positive effects for the groomers, it does not take place in a social vacuum. Individuals in many animal species monitor group members' mating behaviour [53–55], police aggressions between others [56–58], and third parties reconcile or console aggressors to reduce the negative impact of interactions on their own fitness [59–61]. Groomers are surrounded by other group members, for whom the grooming interaction potentially has negative consequences, posing the question whether they actively seek to influence it to their own advantage.

Grooming between two individuals can negatively affect bystanders in at least three ways. Owing to time constraints, grooming between two individuals may influence their availability to others, but also how the groomers will allocate other services like feeding tolerance or support during aggressive interactions. Thus, groomers may restrict bystanders in their attempt to maximize their own benefits [38,45]. Secondly, grooming can negatively impact bystanders if an alliance between the two groomers could threaten the rank or access to resources for the bystander (i.e. ‘bridging alliance’ or ‘revolutionary alliance’[45,62]). This is particularly relevant if an individual close in rank to the bystander grooms with someone of higher rank. Primates have anecdotally been described to block alliance formation of individuals with similar rank [63,64], while in ravens, individuals disrupt the formation of new social bonds which influence the intervener's own rank [65]. Thirdly, grooming can negatively influence a bystander by jeopardizing the resources (e.g. grooming time, coalitionary support) the bystander has invested in one of the groomers in the past. If this individual now grooms with someone else, there is a threat that they might defect, decreasing the services they can reciprocate, and making their support for the bystander less predictable. Losing a social partner due to death has far-reaching consequences on the behaviour, hormone levels and health of individuals [66], but the impact of partner defection is unclear. Feral horses intervene to stop bond partners from affiliating with other group members, potentially to prevent defections [67,68]; relationship protecting behaviour has been shown experimentally in dogs [69].

Because of these three reasons, bystanders are predicted to not passively observe, but rather to actively alter the outcome of grooming interactions in their favour. Importantly, countering the negative impacts of threatening alliance formation or affiliation partner defection requires bystanders to keep track of and evaluate grooming between other group members and to then act upon dominance rank and social relationship information. To test how bystanders influence grooming interactions, we use direct observations of grooming interventions, i.e. *any behaviour of a bystander directed at one or both groomers that could change the ongoing grooming bout*. Interventions into affiliative interactions have been shown to effectively reduce subsequent affiliations [70]. In primate and non-primate studies, when grooming interventions were considered, the focus has often been on bystanders supplanting one of the groomers and gaining access to the other [39,43,71,72], or on the disruption of the affiliative interaction [65,67–69]. Our definition is broader than previous studies on grooming interventions [65,67,69,72], as our focus is on the behavioural decision of the bystander to approach the grooming dyad, rather than on the decision of the groomers to stay or leave as in previous studies. An approach can result in the following outcomes: both groomers cease to groom (disruption or ‘separating interventions’ [63,64]), one leaves and one remains (supplant), or they continue grooming, with the intervener joining and turning the bout into a polyadic grooming session [73]. In the latter case, there is a chance of remaining as the sole grooming partner of one groomer after a time delay. While joining a grooming bout does not always immediately disrupt the original grooming interaction, it allows the intervener to offset the negative impact of the original bout by increasing their own grooming time and reducing the time a competitor spends alone with an attractive grooming partner.

We studied two sympatrically living species of non-human primates, sooty mangabeys (*Cercocebus atys atys*) and Western chimpanzees (*Pan troglodytes verus*), in the Taï National Park, Côte d'Ivoire, as part of a project investigating the link between social complexity and cognitive abilities in these two species. Both have large multi-male, multi-female social groups [46,74] where individuals compete for grooming partners. In sooty mangabeys, grooming is focused on individuals close in rank, with indications that low-ranking groomers (LRG) invest more than high-ranking ones [2]. Grooming supplants have been shown to be directed mainly down the hierarchy, with the LRG being more likely to leave [72]. There are mixed results concerning grooming distribution between and even within different chimpanzee communities [75], with some authors finding a preference for higher-ranking and closely ranked individuals [39,42], while others did not find a rank effect [76]. No bias towards closely ranked or HRG partners was previously found in Taï chimpanzee females [77]. While grooming supplants exist [39], chimpanzees regularly groom polyadically, which is rare in other primates [73]. We chose these two species because they differ in a number of variables that are likely to influence grooming interventions. They differ markedly in their tolerance around and monopolizability of resources: sooty mangabeys display strong within-group contest competition [46,78] and highly linear, steep dominance hierarchies in both sexes [2,46] where rank usually defines the outcome of competition (98% of aggressions down the hierarchy in females, 88% in males; A. Mielke 2017, unpublished data). Adult male mangabeys do not groom each other. Male and female chimpanzees show linear hierarchies, with differences across groups in hierarchy steepness [42,79,80]. In our communities, 82% of male and 63% of female aggression were directed down the hierarchy (A. Mielke 2017, unpublished data), indicating moderate hierarchy steepness, which corresponds to moderate reproductive skew in this population [81]. Chimpanzees are relatively tolerant around resources (even though aggression is more likely in a feeding context [79]), and regularly share food [26,82,83]. Alliances of subdominant individuals are able to monopolize resources against dominant individuals [63]. Thus, in both species, high-ranking individuals are attractive partners who can confer benefits for grooming, and they can monopolize grooming partners, but this effect is less pronounced in chimpanzees than in mangabeys. Chimpanzees live in a social system with high fission–fusion dynamics [84]. While sooty mangabeys do not exhibit high levels of fission–fusion dynamics and the group usually travels as a cohesive whole, individuals are not constantly in visual contact with each other owing to the spread of the group within their forest habitat (A. Mielke 2017, personal observation). Thus, in both species, bystanders differ from grooming bout to grooming bout.

We predict that species differences in the impact of rank on resource monopolization will influence grooming interventions, with chimpanzee interventions being less defined by the ranks of groomers and bystanders. In chimpanzees, the difference in attractiveness of low- and high-ranking individuals should be less pronounced than in mangabeys, where high-ranking individuals can offer stronger benefits in return for grooming. Thus, we predict that mangabeys should intervene more to gain access to high-ranking individuals than chimpanzees. Priority of access in mangabeys, but not chimpanzees, should shape intervention patterns by preventing low-ranking individuals from intervening, also reducing their success rate if they try.

We investigate when bystanders intervene into grooming bouts, which of the groomers they target (i.e. which groomer they attempted to groom themselves), what determines intervention success, and how intervention patterns compare in the two primate species. Our goal was to determine whether interventions function to gain access to attractive grooming partners (pro intervention [85]), or if they are used to counter the negative effects of grooming between group members (contra intervention [85]), which would involve an active monitoring of others' interactions. Note that these two options are not mutually exclusive and different interventions can serve different purposes. In the first case, we predict that intervention likelihood increases if one groomer is a more attractive grooming partner for the bystander, with an increased rank of the HRG or a stronger affiliative relationship with the preferred groomer (PG); also, interveners should target the HRG or PG to increase their own benefits (see table 1 for predictions). If interventions function to impede specific grooming bouts, bystanders will intervene more if a connection between the groomers could threaten their own position or social relationships: to prevent alliance formation of the LRG when they are close to them in rank [45], or if either groomer has a strong affiliative relationship with the bystander [68], but without necessarily targeting the HRG or the PG. We also predict that they intervene more when the groomers are not affiliated strongly with each other, to prevent alliance formation [65].

**Table 1.** Summary of predictions.

predictions	model	outcome
*grooming interventions give interveners access to attractive grooming partners*		
(1) grooming interventions are more likely when HRG is high-ranking or close to bystander in rank	1.1	not supported
(2) grooming interventions are independent of LRG rank	1.1	not supported
(3) grooming interventions are more likely when PG has strong affiliative relationship with bystander	1.2	supported
(4) grooming intervention likelihood is not affected by affiliative relationship between groomers	1.2	not supported
(5) interveners target HRG	2	not supported
(6) interveners target PG	2	not supported
(7) intervention success independent of affiliative relationships	3.2	supported
*grooming interventions impede grooming with potentially negative consequences for bystander*		
(1) grooming interventions are independent of HRG rank	1.1	supported
(2) grooming interventions are more likely when LRG is close to bystander in rank	1.1	supported
(3) grooming interventions are more likely when PG has strong affiliative relationship with bystander	1.2	supported
(4) grooming interventions are more likely if groomers have a weak affiliative relationship	1.2	supported
(5) interveners do not target HRG	2	supported
(6) interveners do not target PG	2	supported
(7) intervention success is independent of affiliative relationships	3.2	supported
*grooming interventions are affected by the social system of the species*		
(1) in mangabeys, high-ranking bystanders are more likely to intervene than low-ranking bystanders	1.1	supported
(2) in mangabeys, high-ranking interveners are more likely to be successful than low-ranking interveners	3.1	supported
(3) in mangabeys, but not chimpanzees, individuals intervene to gain access to high-ranking groomers	1.1	not supported
(4) in chimpanzees, but not mangabeys, bystanders intervene more when their affiliative partners are grooming	1.2	not supported
(5) in chimpanzees, low- and high-ranking bystanders are equally likely to intervene	1.1	supported
(6) in chimpanzees, low- and high-ranking interveners are equally likely to be successful	3.1	supported

## 2. Material and Methods

### 2.1. Data collection

Data on grooming interventions were collected in Taï National Park, Côte d'Ivoire [74] from 2013 to 2015, using half- and full-day focal animal sampling [86]. Two observers (A.M., L.S.; inter-observer reliability greater than 90%) recorded activities and all social behaviour of male and female chimpanzees above 12 years of age in the ‘south’ (habituated since 1997; A.M., L.S.) and ‘east’ (habituated since 2006; L.S.) communities and adult (above 5 years) sooty mangabeys (habituated since 2013; A.M.; table 2). To reliably determine dominance hierarchy and social bonds, we augmented our dataset with full-day focal observations of grooming, aggressions, proximity, pant grunts and supplants collected by trained observers (A.M., J.F.G., A.P., L.S.) and taken from the Taï Chimpanzee Project's long-term database (south: 1999–2016; east: 2007–2016; mangabeys: 2014–2016). Data were only included once observers had more than 80% overlap with a trained reference observer when collecting data simultaneously.

**Table 2.** Characteristics of the study groups, observation time, grooming interactions, and interventions.

	focal individuals				interventions	
	male	female	observation hours	grooming interactions	male	female
mangabey	7	20	728 h	1209	28	110
chimpanzee south	5	6	1991 h	1343	155	110
chimpanzee east	5	11	1384 h	1067	72	75

We included two chimpanzee communities to get an indication of whether effects are group-specific. Data were collected following the same protocol in both species, using customized CyberTracker data collection software (CyberTracker Conservation 2013). We recorded dyadic and polyadic social interactions within focal observations. In addition, we recorded all occurrences of grooming interactions in the party [86] that were visible to the observer (and, implicitly, the focal). As chimpanzees, but not mangabeys, have high fission–fusion dynamics, we used different measures to define bystanders of a grooming bout. In chimpanzees, individuals who were within visual range of the focal (usually within a range of less than 50 m, average around 30 m) were recorded continuously and constituted the bystanders of a grooming bout. This is in line with the definition of ‘party’ used in different chimpanzee study sites [51,77,87]. For mangabeys, we recorded all individuals that appeared in visual range during a 5 min period, and considered these the bystanders for grooming of the focal in this time period. As both species spend the majority of their time on the ground, especially when travelling, and party composition is relatively stable, this approach allowed us to identify all potential interveners with relatively high certainty.

Interventions were defined as any behaviour of a bystander directed at one or both of the groomers that could potentially change the course of a grooming bout. We included grooming interactions and interventions (both focal and all occurrence) into the dataset if all three individuals involved were focal individuals during this study (to assure that enough focal data were available to reliably calculate dominance rank and affiliative relationship values) and if all of the following were available: identity of groomers, duration of the bout, identity of all bystanders present that could potentially intervene, the amount of time they were present, type of intervention (disruption, supplant or joining), and the outcome of the intervention. We considered consecutive dyadic grooming interactions as part of the same bout if they involved at least one of the same groomers and started within 5 min of the end of the last grooming interaction. We treated polyadic grooming bouts as multiple dyadic interactions. If an individual intervened multiple times into the same grooming dyad during the same bout, we only considered the first attempt, but multiple bystanders could intervene in the same grooming dyad.

### 2.2. Dominance ranks

Hierarchy ranks of all communities were calculated dynamically, using a modification of the Elo rating method [88,89] as proposed by Foerster *et al*. [80]. We used unidirectional pant grunt vocalizations (given by the lower-ranking of two individuals [90]) in chimpanzees, using all available data for individuals above the age of 9 or after they were orphaned, from 1999 to 2017 in the south community (8391 pant grunts between males, 846 between females), and from 2007 to 2016 in the east community (3584 pant grunts between males, 195 between females). For both chimpanzee communities, we calculated ranks within males and females separately and afterwards combined the results, as all males are higher-ranking than all females in this field site [4]. For sooty mangabeys, females regularly supplant younger males (A. Mielke 2017, unpublished data), so one common hierarchy was established for both sexes. We used non-aggressive supplants in sooty mangabeys (given by the higher-ranking of two individuals [46]) to establish hierarchies (2909 supplants) between all individuals above 3 years of age.

While the original Elo rating tracks the winning likelihood of one individual over the other using a fixed start value and change factor *k*, the modification by Foerster *et al*. uses maximum-likelihood estimation to optimize the *k* and allow individuals to enter the hierarchy with different start values. This reduces the need for a burn-in phase where ranks are relatively uncertain, does not assume rank changes where none exist, and reduces the need for *a priori* decisions by the researcher. However, it shows some problematic properties when facing sparse datasets, as we see for female chimpanzees in the communities studied here. We, therefore, added a further modification to the script provided by Foerster *et al*. [80]: rather than estimating the *k* and start values of the Elo rating by optimizing only the winning likelihood, which might underestimate the size of the *k* in sparse datasets and, therefore, miss rank changes, we additionally optimized the number of correct classifications (i.e. interactions where the higher-ranking individuals wins and the lower-ranking individual loses) by varying the number of iterations of the optimization algorithm and selecting the solution that maximizes the number of correct classifications (A. Mielke 2017, unpublished data). Assuming that the chosen interaction type is overwhelmingly unidirectional (as has been suggested for chimpanzee pant grunts [79,90] and mangabey supplants [46]), this modification prevents apparent rank changes that are based on the interactions of one individual with a third group member, and it also improves the detection of rank changes of dyads that rarely interact. Ordinal ranks were standardized between 0 and 1, with 1 being the highest-ranking individual on any given day.

### 2.3. Social relationships

To account for the fact that affiliative relationships between individuals might change over time, we adopted a method similar to Elo ratings, the dynamic dyadic sociality index (DDSI [91]), to calculate dyadic affiliation strength using data collected in the three communities between January 2012 (January 2014 for the mangabeys) and May 2015. The DDSI has the advantage of allowing researchers to represent relationships between two individuals on a daily basis based on their past positive and negative social interactions, thereby avoiding decisions about appropriate time periods to aggregate data. We used the duration of grooming exchanges (mutual grooming duration counted twice, as grooming given and grooming received), 1 m proximity during resting and feeding (using the duration of contact in chimpanzees, where this information was available, while using proximity as events during activity changes for the mangabeys), and aggression events (with multiple aggressions of the same dyad and direction within 10 min of each other counting as one) [92,93]. All dyads enter at a value of 0.5, either from the beginning of the time period or when individuals join the group. From there, positive interactions (grooming, proximity) change the value of the dyad upwards, and negative interactions (aggressions) downwards, with the weight of the interaction being determined by its frequency in the dataset, thus giving rarer interactions a stronger impact on the index. Interactions for which duration was considered were entered into the DDSI after standardizing durations by the median duration of any interaction of this kind. Thus, if grooming is twice as likely as aggressions, then a grooming interaction of twice the median duration ‘compensates’ for one aggression. As in Elo ratings [89], ‘expected’ interactions (e.g. positive interactions between individuals with a high value) have a weaker impact on the index than ‘unexpected’ interactions. While the value of the dyad is increased/decreased, the value of both interactors with all other community members are decreased/increased, respectively, so that the average index of all dyads remains at 0.5 throughout. Thus, the index has a direct interpretation: dyads with values above 0.5 had more positive than negative interactions in the past, or they engaged in many negative interactions with other group members but not this one; dyads with values below 0.5 either had more negative than positive interactions or had positive interactions with other group members. For dyads to have consistently high values, they need to continuously invest in each other. The DDSI value of any dyad is extracted for the day before grooming interactions used in the dataset, to make the relationship value and the grooming bout independent from each other.

### 2.4. Models and statistical analysis

We fitted multiple models to test the impact of dominance ranks and affiliative relationships on grooming interventions (see the electronic supplementary material, table S1 for model parameters). All models were generalized linear mixed model (GLMM) with binomial error structure and logit link function [94], implemented with R statistical software [95] using the package lme4 [96]. For all models, we included the sexes of the two groomers and the bystander as fixed effect control predictors. In model 1, where sufficient data were available, sexes of the groomers and bystanders were included in interaction with group identity, as the impact of the sexes on intervention likelihood is predicted to differ between species. In models 1.1 and 1.2, we controlled for the presence of female groomers that were maximally tumescent or with an infant younger than 3 months, as these have been shown to influence grooming behaviour [33,34]. We entered a variable ‘reproductive state of groomers’ with the values 1 (at least one of the two groomers is maximally tumescent or with an infant) or 0; though the two reproductive states are likely to attract different interveners, the complexity of the models and low frequency of both prevented us from testing their impact directly. As grooming interactions often last considerable time periods, it was not possible to include bystander distances or grooming direction, as bystanders move around and grooming directions change, even though it is possible that closer bystanders are more likely to intervene. As comprehensive kinship data for adults were only available for the south community, we could not test the impact of kinship on interventions. For each model, we conducted full null model comparisons using a likelihood ratio test [97], where the null model included only the control predictors and group identity and the same random effect structure as the full model, to test whether the test predictors collectively had a significant effect.

Model 1 tested which factors influenced the likelihood that a bystander would intervene into a grooming bout. For this, we analysed every combination of each dyadic grooming interaction (in 2012 individual grooming bouts) with each bystander for this interaction, comparing whether intervening (*n* = 550) differed from non-intervening bystanders (*n* = 20 656). Owing to model complexity, we fitted two separate models.

Model 1.1 tested the impact of dominance ranks on intervention likelihood. To test whether an attraction to higher-ranking individuals existed in either group, while accounting for the effects of both rank and rank distances between individuals, we included a three-way interaction between the rank of the HRG, the rank of the bystander, and group identity as a test parameter. The three-way interaction accounts for rank positions and rank distance between the individuals. To test whether bystanders intervened to influence grooming of close-ranking competitors, or whether groomer rank restricted access, we entered the three-way interaction between bystander rank, the rank of the LRG, and group identity as test parameter. We controlled for the dyadic relationship scores of all three dyads.

In model 1.2, we tested the impact of affiliative relationships of the bystander, with the groomer with whom the bystander had the higher DDSI score (PG) and with whom they had the lower DDSI score (‘non-preferred groomer’, NPG), on intervention likelihood. To test for the impact of affiliative social relationships in both species, we included the three-way interaction between the relationship score of bystander and PG, the relationship score of bystander and the NPG, and group identity as test predictor. To test for group differences in the impact of the groomers' affiliative relationship, we entered a two-way interaction between group identity and the DDSI score of the groomers as a test predictor. We controlled for the rank values of all three individuals. We included the log-transformed presence time of each bystander during the grooming bout as an offset term into models 1.1 and 1.2 to account for differences in the opportunity to intervene in grooming bouts of different durations [98], and for the fact that not all bystanders are present continuously. We included the identity of the grooming bout in models 1.1 and 1.2 to account for the fact that grooming bouts were represented by multiple non-independent entries.

In model 2, we analysed whether intervening individuals preferably target HRGs or their preferred partners. We used only interventions in which the target (the first grooming partner after the intervention) could be determined (*n* = 456), thereby excluding interventions that did not lead to grooming by the intervener. To be able to control for non-independence of data and retain the random effects structure, but still test the impact of the fixed effects on individual choice, we used a ‘repeated measures’ design [14]. Every intervention was included into the dataset using two data points, one representing the target with the binary variable ‘choice’ as 1, and one presenting the non-target with ‘choice’ as 0. As this would leave us with zero variance in the probability of the outcome, we tested the significance of the model using repeated random selection. We ran 1000 selections, each containing one randomly chosen data point per intervention event. For each selection, we fitted a GLMM to determine the coefficients and compared the full and null model using likelihood ratio tests. We report the mean *χ*^2^, parameter estimates, standard errors, *z*-values and *p*-values across the 1000 models as the result. Test predictors for this model were: the three-way interaction of the dominance rank of the target with that of the intervener and group identity, to test whether individuals in either species targeted higher-ranking or closely ranked groomers; and the two-way interaction of group identity with the DDSI of the intervener with the target, to test whether individuals in either species targeted the groomer they were more affiliated to. We controlled for grooming direction at the time of intervention.

In model 3, we tested what determined intervention success, again focusing on interventions where the target of the intervention was known. Interventions were ‘successful’ if the intervener gained access to a groomer (supplant) or subsequently received grooming from or remained as the sole grooming partner of either groomer (joining). It was not successful if the two groomers continued grooming, or if the intervener joined but was ignored by the groomers and left without receiving grooming. Of the 462 interventions with a known outcome and target, 271 were successful. Owing to model complexity, we divided the model into models 3.1 and model 3.2. In model 3.1, we included the three-way interaction of the intervener rank, group identity and the rank of the non-target, to test whether the impact of rank relations on intervention success differed between the two species. We also included the three-way interaction between the two groomer ranks and group identity, to test whether it is easier to intervene into grooming between individuals with higher rank distance. In model 3.2, we tested whether interveners in either species succeeded more if they were more affiliated with either groomer, or if the two groomers were affiliated less with each other, by including two-way interactions between the relationship scores of intervener and each groomer with group identity, and the interaction between the relationship score of the groomers with group identity.

We included the three identities of bystander and groomers (55 individuals) and the three dyad combinations between them (513 dyads) into models as random effects, to account for individual and dyadic differences in intervention behaviour and the fact that not all individuals and dyads were equally likely to be observed grooming [98]. We tried to include all possible random slopes of fixed within random effects to keep type I error rate at the nominal level of 5% [99,100]. However, as this would have increased model complexity beyond what the data could support, we included only random slopes of the test predictors within the three individual identities in each model [99]. Quantitative variables were *z*-standardized to a mean of zero and a standard deviation of one [101]. We tested significance of the interactions, lower-order interactions, and main effects by systematically dropping them from the model one at a time [97] and comparing the resulting model with the full model using the ‘drop1’ function in R [95]. Variance inflation factors (VIF) [102] were derived using the function vif of the R-package ‘car’ [103] applied to a standard linear model excluding the random effects and the interactions for each of the models. Collinearity was not an issue in any of the models (maximum VIF = 3.845).

## 3. Results

### 3.1. When do bystanders intervene (model 1)?

**Figure 1.**
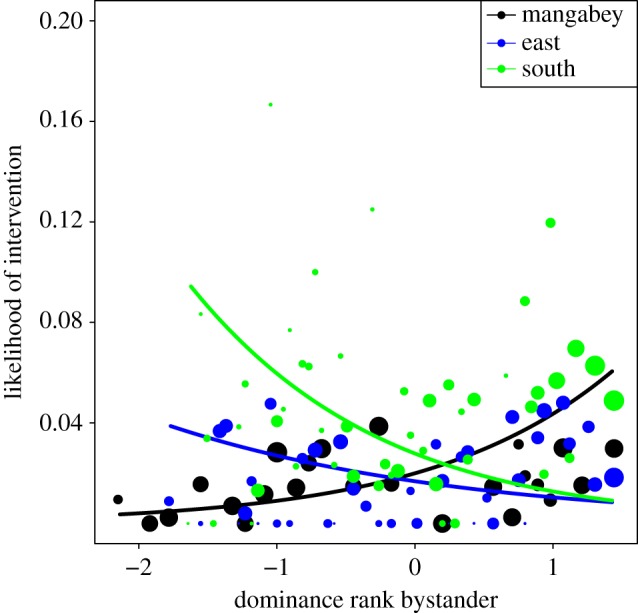
The probability of the bystander to intervene depending on the effects of the interaction of bystander rank (*z*-standardized, original mean = 0.614, s.d. = 0.263) with group identity (model 1.1). Higher values on the x-axis depict high bystander ranks. Mangabeys (black) show a significant positive effect with increasing rank, while south (green) shows a significant negative effect, and east showed no effect. Shown are the observed probabilities to intervene into a grooming bout of average duration (larger point areas (range 1–639 observations) denote a larger number of observations) as well as the model results (lines).

**Figure 2.**
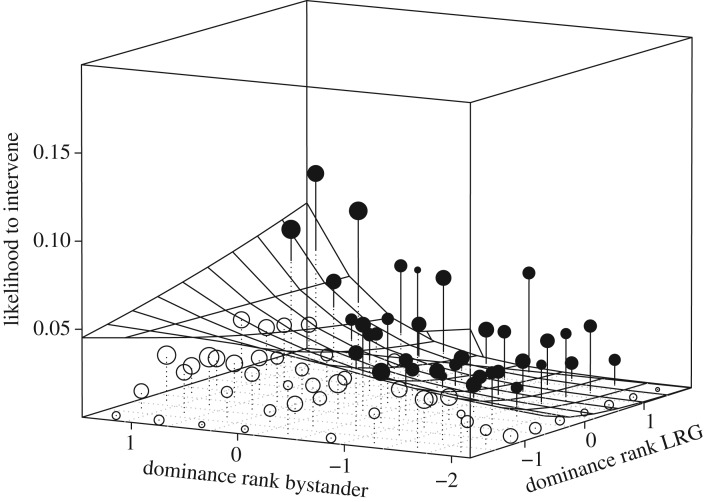
The probability of the bystander to intervene depending on the effect of interaction of bystander dominance rank (*z*-standardized, original mean = 0.614, s.d. = 0.263) and dominance rank of the LRG (*z*-standardized, original mean = 0.515, s.d. = 0.248; model 1.1). High values on the axes depict high individual rank. Shown are the observed probabilities to intervene into a grooming bout of average duration (larger point volumes (range 3–1124 observations) denote a larger number of observations) as well as the model results (surface). No group differences were observed.

The full null model comparison for model 1.1 showed a significant impact of the test predictors (likelihood ratio test: *χ*^2^_15_ = 30.23, *p* = 0.011; electronic supplementary material, table S2a). None of the three-way interactions were significant. After they were removed, the interaction between group identity and bystander rank was significant (*χ*^2^_2_ = 19.42, *p* < 0.001; figure 1; electronic supplementary material, table S2b). The estimates of the main effects when using each group as reference revealed that in mangabeys, higher-ranking bystanders had a higher likelihood to intervene than lower-ranking bystanders (estimate = 0.92, s.e. = 0.24, *p* < 0.001), while south showed the opposite effect, with LRGs having a higher likelihood to intervene (estimate = −0.78, s.e. = 0.29, *p* = 0.008). East showed no effect (estimate = −0.42, s.e. = 0.29, *p* = 0.142). The interaction between bystander rank and the rank of the LRG was significant in all three groups (*χ*^2^_2_ = 5.50, *p* = 0.019). High-ranking bystanders intervened more if the LRG was itself high-ranking. Low-ranking bystanders intervened more when the LRG was also low-ranking (figure 2). None of the interactions with the rank of the HRG or its main effect were significant. For the control variables, a significant effect of HRG sex revealed interventions were more likely when the HRG (*χ*^2^_1_ = 7.20, *p* = 0.007) and the LRG (*χ*^2^_1_ = 10.49, *p* = 0.001) were male.

**Figure 3.**
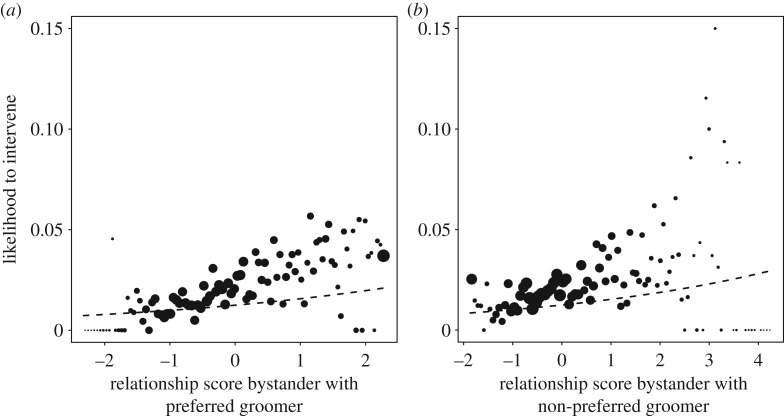
The probability of the bystander to intervene depending on the effects of *z*-standardized DDSI relationship scores between the bystander and the PG (*a*; original mean = 0.565, s.d. = 0.099), and the bystander and NPG (*b*; original mean = 0. 468, s.d. = 0.073). Shown are the observed probabilities to intervene into a grooming bout of average duration (larger point areas denote a larger number of observations (range 1 to 888 observations)) as well as the model results (model 1.2, lines). No group differences were found for either predictor.

**Figure 4.**
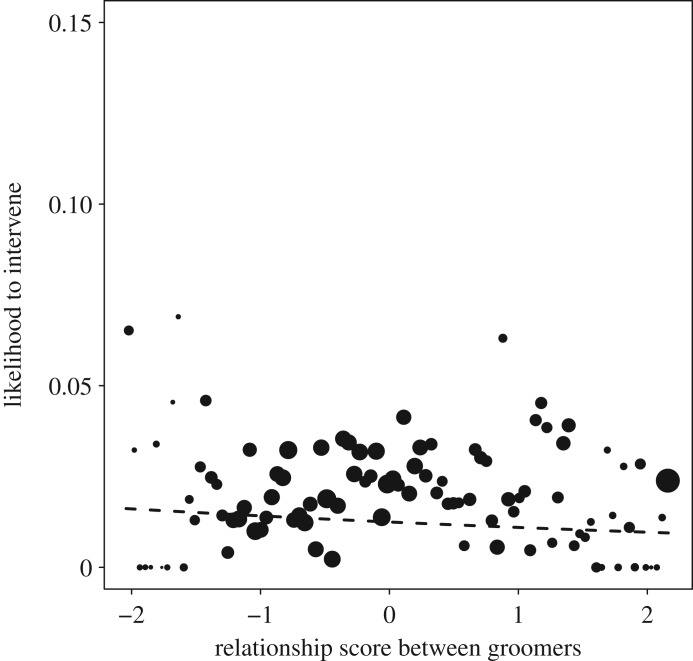
The probability of the bystander to intervene depending on the effects of *z*-standardized DDSI relationship scores between the two groomers (model 1.2; original mean = 0.553, s.d. = 0.111). Shown are the observed probabilities to intervene into a grooming bout of average duration (larger point areas denote a larger number of observations (range 1 to 915 observations) as well as the model results (lines). No group differences were found.

The full null model comparison for model 1.2, focusing on affiliation strengths, revealed that the test predictors had a significant impact on the response (*χ*^2^_12_ = 26.96, *p* = 0.008; electronic supplementary material, table S3a). After removing non-significant interaction terms, it became clear that the intervention likelihood in all communities was higher with higher relationship scores between bystander and both the PG (*χ*^2^_1_ = 7.63, *p* = 0.013; figure 3*a*; electronic supplementary material, table S3b) and the NPG (*χ*^2^_1_ = 3.70, *p* = 0.001; figure 3*b*). Additionally, in all groups there was a lower likelihood to intervene when relationship scores between the groomers were high (*χ*^2^_1_ = 3.65, *p* = 0.001; figure 4). Looking at the control variables, intervention likelihood in all groups was higher if one of the groomers had a swelling or new-born infant (*χ*^2^_1_ = 9.20, *p* = 0.002) or the PG (*χ*^2^_1_ = 9.18, *p* = 0.003) or NPG (*χ*^2^_1_ = 5.89, *p* = 0.015) was male.

### 3.2. Which groomer was targeted (model 2)?

The full null model comparison, testing which groomer was targeted during interventions, revealed no significant impact of the rank or affiliative relationship variables when controlling for identities and group (*χ*^2^_11_ = 12.68, *p* = 0.382; table 3; electronic supplementary material, table S4).

**Table 3.** Intervention target choice in the three communities, based on whether they chose the higher-/lower-ranking of the two groomers, or the one they are more or less affiliated with (*n* = 462).

	target HRG	target LRG	target PG	target NPG
mangabey	44	29	48	25
chimpanzee east	61	72	58	75
chimpanzee south	138	118	134	122

### 3.3. What determined intervention success (model 3)?

**Figure 5.**
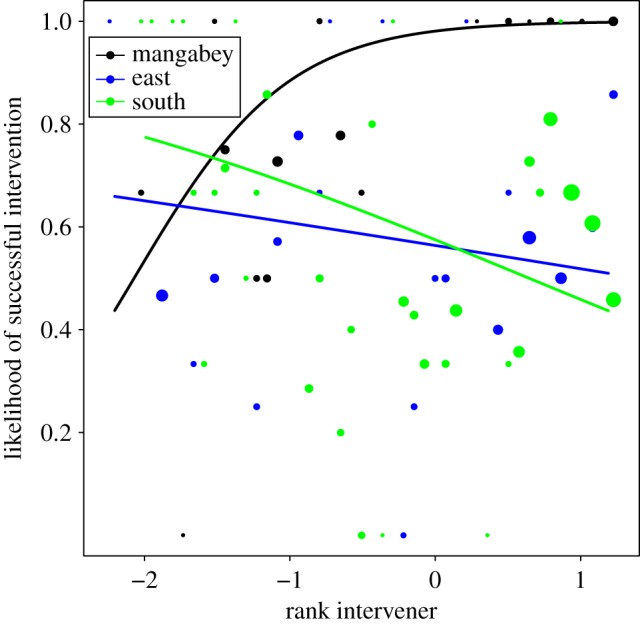
The probability of the intervener to intervene successfully depending on the effects of the interaction of the intervener's rank (*z*-standardized, original mean = 0.693, s.d. = 0.241) with group identity (model 3.1). High values on the *x*-axis depict high intervener rank. Mangabeys (black) show a significant positive effect, with high-ranking individuals being more likely to successfully intervene. Neither chimpanzee community showed a significant result. Shown are the observed probabilities to successfully intervene into a grooming bout (larger point areas denote a larger number of observation (range 1 –30 observations) as well as the model results (lines).

There was a species difference in the intervention outcomes (table 4): mangabeys interventions almost exclusively led to one or both groomers leaving (supplants or disruptions). In both chimpanzee communities, bystanders mainly joined grooming bouts, making them polyadic. The full null model comparison of model 3.1 revealed a significant effect of the rank variables on intervention success (*χ*^2^_15_ = 27.68, *p* = 0.024; electronic supplementary material, table S5a), with mangabey interveners of high rank being significantly more likely to succeed, which was not observed in either chimpanzee community (*χ*^2^_2_ = 8.58, *p* = 0.014; figure 5; electronic supplementary material, table S5b). There was a trend for interventions in both species to be more successful when the rank of the non-target decreased (*χ*^2^_1_ = 3.80, *p* = 0.051), and male interveners were more successful than females in both species (*χ*^2^_1_ = 4.78, *p* = 0.029).

**Table 4.** Intervention types, success of interventions (gaining access or ending grooming bout), and rank relationships of the focal towards both groomers in mangabeys, south chimpanzees and east chimpanzees in cases where intervention types were known (*n* = 515).

		intervener higher- ranking than both		intervener of intermediate rank		intervener lower- ranking than both	
	intervention type	successful	unsuccessful	successful	unsuccessful	successful	unsuccessful
mangabey	supplant	26	0	25	1	2	0
	disrupt	23	2	14	0	3	0
	join	2	7	1	5	1	3
	total	51	9	40	6	6	3
chimpanzee east	supplant	1	0	3	0	2	0
	disrupt	0	0	0	0	0	1
	join	13	8	27	20	26	33
	total	14	8	30	20	28	34
chimpanzee south	supplant	6	1	9	1	3	1
	disrupt	3	0	2	0	2	0
	join	25	25	46	33	52	57
	total	34	26	57	34	57	58

Model 3.2 was non-significant (*χ*^2^_9_ = 5.58, *p* = 0.782; electronic supplementary material, table S6), indicating that bond variables did not determined how successful the intervention was.

## 4. Discussion

In this study, we investigated the influence of dominance rank and social relations between bystanders and groomers on three outcome variables: (i) whether a grooming intervention occurred, (ii) who the target of the intervention was, and (iii) intervention success. We found that rank and affiliative relationships affected grooming intervention behaviour in both sooty mangabeys and chimpanzees. We argue that, while they differed in who could intervene, the motivation to intervene was similar in all three groups and across both species. Interventions seemed to occur mainly when bystanders could gain from impeding grooming of that dyad, indicating that both species alter grooming interactions between others to their advantage.

While it is hard to identify species differences when comparing only three communities, we nonetheless found some persistent differences distinguishing the mangabey group from both chimpanzee communities. The lack of polyadic grooming in mangabeys limited who could intervene and how. Aggression in the intervention context was rare (around 4% of interventions) and of low intensity in both species, so most interventions initially involved one individual approaching a grooming dyad. In the mangabeys rather than the chimpanzees, bystanders higher in rank than at least one groomer were more likely to approach and one or both groomers would react by abandoning the grooming bout, as previously reported [72]. Also, high-ranking interveners were more likely to be successful. By contrast, in the two chimpanzee communities, where polyadic grooming was common, low-ranking individuals could both intervene and be successful in their interventions at least as often as high-ranking individuals. In the south community, low-ranking individuals were even more likely than high-ranking individuals to intervene, potentially because the negative impact of grooming affects them stronger, or because high-ranking individuals are more likely to already groom somebody. The only indication for an impact of rank on intervention success in chimpanzees was a trend for increased likelihood of success if the non-target, i.e. their competitor, was of low rank. Unlike mangabeys, high-ranking chimpanzees are, therefore, less able to monopolize grooming partners, making success less predictable and giving groomers more flexibility in continuing to groom whom they choose. As the grooming partner as a resource in mangabeys is limited, high-ranking individuals will have priority of access, restricting low-ranking individuals. In chimpanzees, where the resource is not limited (multiple individuals can groom the same individual), rank will not be the only determining factor in competition. Polyadic grooming is possible because individuals are able to remain in such close distances for long periods of time without aggression, owing to the tolerance in the species.

The factors motivating bystanders to intervene seem to be similar in mangabeys and chimpanzees. Interventions were not more likely in any group if the HRG was high in rank or close in rank to the bystander, as would be predicted if interventions were a way to gain access to attractive grooming partners. However, individuals in all groups were more likely to intervene if the lower-ranking (but not the higher-ranking) groomer was close to them in rank. This would fit a scenario where bystanders intervene to impede alliance formation of competitors who are close to them in rank, as the HRG could provide coalitionary support as a return for the grooming [45,64]. The focus on closely ranked competitors in chimpanzees exists despite the fact that joining interventions do not always disrupt the ongoing grooming immediately; however, joining appears to restrict the time that others groom, limiting competition for potential coalition partners and thus also limiting the negative impact on the bystander's own social environment. Also, joining interventions create a situation where one individual can choose directly between two grooming partners, and the choice might increase the predictability of future support.

In both species, bystanders intervened more when they had a strong affiliative relationship with one or both groomers, which could be evidence for an attraction to affiliation partners [44]. However, interveners did not preferentially target these affiliated individuals, and intervention success was independent of their relationship score. In mangabeys, who only groomed one of the groomers after successful interventions, we would predict that only the relationship score with the PG would be significant if the goal of the intervention was to gain access to friends. However, the relationship scores of the bystander with both groomers had a significant impact on their likelihood to intervene, while not reducing or increasing intervention success. Feral horses' interventions into others' affiliations have been interpreted as prevention of the defection and bond formation of preferred partners [68], and a similar explanation seems likely for the similar patterns we find in both our study species. Interveners prevent defection of the partner and protect their relationships, by disrupting the partner's grooming or by participating in the bout. However, alternative explanations could apply, especially for the polyadic grooming of chimpanzees. The presence of a bond partner may enable the intervener to access individuals that are usually out of its reach and open a wider social network, which may explain why the PG is not always the target of grooming intervention. Individuals could change the grooming partner throughout the polyadic bout. Alternatively, sharing a grooming partner, like the sharing of food resources [83], could be a bonding behaviour. More detailed analyses of polyadic grooming in different species are needed to understand its function.

Mangabey and chimpanzee bystanders intervened more when the relationship score between the two groomers was low. The relationship score between groomers had no obvious effect on intervention success, indicating that the lower intervention rate was not a result of affiliated groomers being harder to separate. Interventions might impede the formation of new alliances rather than restrict existing stable bonds, as reported for ravens [65]. Bystanders differentially intervened based on the affiliative relationship between the groomers, supporting other studies showing that primates possess triadic awareness [104–107]. This ability involves keeping track of affiliative relationships between others, which, unlike dominance relationships, most likely cannot be assessed by any single behavioural metric [104].

We provide quantitative evidence that grooming interventions could function to reduce the negative impact for the bystander of others' grooming in two primate species. Bystanders used grooming interventions towards dyads whose grooming could threaten their rank position or relationships, particularly dyads of close-ranked competitors, own affiliation partners and emerging alliances. While similar patterns have been found in other species [65,68], this study is, to our knowledge, the first to combine dominance rank and affiliative relationships of all three participants, and also evaluate the outcome of the intervention. Our results indicate that individuals monitor grooming interactions, make a multivariate assessment (based on ranks, their own relationships and the relationships between groomers) of whose alliances could threaten them, and potentially use this information to manipulate third-party social interactions and bond formation. It has been indicated that primates use policing, third-party reconciliation, contra interventions and consolations to positively influence social relationships between other group members [57,60,85,108–110]; grooming interventions could play the opposite role, by disrupting alliance and bond formation that might otherwise jeopardize one's own social position. Controlled tests are needed to investigate how cognitively complex such behaviours are, and how they are connected to the social emotion of jealousy [69]. Our results indicate that Western chimpanzees' ability to groom polyadically, possibly enabled by relatively high levels of tolerance, allows low-ranking individuals more flexibility in influencing grooming competition than individuals of similar rank in sooty mangabeys, but both showed multivariate intervention strategies. Bystanders take an active interest in grooming bouts they observe, and manipulate grooming of other group members to mitigate the negative impact it could have on their own social life, adding an additional political dimension to grooming competition and triadic awareness.

## Supplementary Material

Model Results Mielke et al

## Data accessibility

Grooming interaction and intervention data for all models, and model scripts available at http://dx.doi.org/10.5061/dryad.2sg0m [111].
