# Supplementary material for: Bystanders intervene to impede grooming in Western chimpanzees and sooty mangabeys
Source: R Soc Open Sci. 2017 Nov 8;4(11):171296. doi: 10.1098/rsos.171296 (PMC5717689; doi:10.1098/rsos.171296)
Supplement: Model Results Mielke et al [file rsos171296supp1.doc]

**Royal Society Open Science**

**Bystanders intervene to impede grooming in Western chimpanzees and sooty mangabeys**

**Alexander Mielke^1,2^, Liran Samuni^1,2^, Anna Preis^1,2^, Jan F. Gogarten^2,3,4^Catherine Crockford^1,2,a^, Roman M. Wittig^1,2,a^**

1 Max Planck Institute for Evolutionary Anthropology, Department of Primatology, Leipzig, Germany

2 Taï Chimpanzee Project, Centre Suisse de Recherches Scientifiques en Côte d'Ivoire, Abidjan, Côte d'Ivoire

3 McGill University, Department of Biology, Montreal, Canada

4 Robert Koch Institute, P3: “Epidemiology of Highly Pathogenic Microorganisms”, Berlin, Germany

a C.C. and R.M.W. contributed equally to this work.

**Electronic Supplementary Material**

**Table S1: Overview of model parameters present in the models fitted (HRG = Higher-ranking Groomer, LRG = Lower-ranking Groomer, PG = Preferred Groomer, NPG = Non-preferred Groomer, DDSI = Dynamic Dyadic Sociality Index)**

| **Model** | **Test Parameters** | **Control Parameters** | **Random Effects** |
| --- | --- | --- | --- |
| 1.1 | Rank Bystander * Rank HRG * Group  Rank Bystander * Rank LRG * Group | DDSI HRG – Bystander  DDSI LRG – Bystander  DDSI Groomers  Sex HRG * Group  Sex LRG * Group  Sex Bystander * Group  Reproductive State Groomers | ID HRG  ID LRG  ID Bystander  Dyad HRG – Bystander  Dyad LRG –Bystander  Dyad Groomers  Bout Number |
| 1.2 | DDSI PG – Bystander * DDSI NPG – Bystander * Group  DDSI Groomers * Group | Rank PG  Rank NPG  Rank Bystander  Sex PG * Group  Sex NPG * Group  Sex Bystander * Group  Reproductive State Groomers | ID PG  ID NPG  ID Bystander  Dyad PG –Bystander  Dyad NPG – Bystander  Dyad Groomer  Bout Number |
| 2 | Rank Target * Rank Bystander * Group  DDSI Target – Bystander * Group | Sex Target  Sex Non-Target  Sex Intervener  Grooming Direction | ID Target  ID Non-Target  ID Intervener  Dyad Target – Intervener  Dyad Non-Target – Intervener  Dyad Groomers |
| 3.1 | Rank Bystander * Rank Target * Group  Rank Bystander * Rank Non-Target * Group | Sex Target  Sex Non-Target  Sex Intervener | ID Target  ID Non-Target  ID Intervener  Dyad Target – Intervener  Dyad Non-Target – Intervener  Dyad Groomers |
| 3.2 | DDSI Target – Intervener* Group  DDSI Non-Target – Intervener* Group  DDSI Groomers * Group | Sex Target  Sex Non-Target  Sex Intervener | ID Target  ID Non-Target  ID Intervener  Dyad Target – Intervener  Dyad Non-Target – Intervener  Dyad Groomers |

Table S2A: Result of **Full Model 1.1**: Impact of dominance rank on bystander likelihood to intervene. Test parameters in bold. Estimate and Standard Error from full model, X², df and p-values result of likelihood ratio test of the reduced model lacking this term with the full model. HRG = Higher-ranking Groomer, LRG = Lower-ranking Groomer, DDSI = Dynamic Dyadic Sociality Index. Full null model comparison: X² = 30.225, df = 15, p = 0.011

| **Term** | **Estimate** | **SE** | **Χ^2^** | **df** | **p** |
| --- | --- | --- | --- | --- | --- |
| Intercept | -6.172 | 0.274 | (1) | (1) | (1) |
| Group East ^(3)^ | -0.254 | 0.628 | (1) | (1) | (1) |
| Group South ^(3)^ | 0.220 | 0.501 | (1) | (1) | (1) |
| Reproductive State Groomers (no=0, yes=1) | 0.242 | 0.151 | 3.779 | 1 | 0.052 |
| Sex HRG (female=0, male=1) | -0.312 | 0.466 | (1) | (1) | (1) |
| Sex LRG (female=0, male=1) | 3.339 | 1.060 | (1) | (1) | (1) |
| Sex Bystander (female=0, male=1) | -0.108 | 0.409 | (1) | (1) | (1) |
| Sex HRG * Group East | 0.899 | 0.866 | 1.597 | 2 | 0.450 |
| Sex HRG * Group South | 0.112 | 0.735 |  |  |  |
| Sex LRG * Group East | -2.886 | 1.157 | 5.483 | 2 | 0.064 |
| Sex LRG * Group South | -2.526 | 1.120 |  |  |  |
| Sex Bystander * Group East | 0.696 | 0.673 | 4.100 | 2 | 0.129 |
| Sex Bystander * Group South | 1.235 | 0.641 |  |  |  |
| DDSI HRG – Bystander ^(4)^ | 0.194 | 0.055 | (2) | (2) | (2) |
| DDSI LRG – Bystander ^(4)^ | 0.153 | 0.053 | (2) | (2) | (2) |
| DDSI HRG – LRG ^(4)^ | -0.175 | 0.053 | (2) | (2) | (2) |
| Rank HRG ^(4)^ | 0.087 | 0.202 | (1) | (1) | (1) |
| Rank LRG ^(4)^ | -0.526 | 0.216 | (1) | (1) | (1) |
| Rank Bystander ^(4)^ | 0.753 | 0.226 | (1) | (1) | (1) |
| Rank HRG * Group East | -0.643 | 0.498 | (1) | (1) | (1) |
| Rank HRG * Group South | -0.209 | 0.455 |  |  |  |
| Rank LRG * Group East | 0.688 | 0.323 | (1) | (1) | (1) |
| Rank LRG * Group South | 0.288 | 0.308 |  |  |  |
| Rank Bystander * Group East | -1.188 | 0.363 | (1) | (1) | (1) |
| Rank Bystander * Group South | -1.516 | 0.368 |  |  |  |
| Rank HRG *Rank Bystander | -0.009 | 0.147 | (1) | (1) | (1) |
| Rank LRG *Rank Bystander | 0.473 | 0.199 | (1) | (1) | (1) |
| **Rank HRG *Rank Bystander * Group East** | 0.402 | 0.284 | 4.572 | 2 | 0.102 |
| **Rank HRG *Rank Bystander * Group South** | 0.273 | 0.295 |  |  |  |
| **Rank LRG *Rank Bystander * Group East** | -0.540 | 0.279 | 3.222 | 2 | 0.200 |
| **Rank LRG *Rank Bystander * Group South** | -0.289 | 0.275 |  |  |  |

1. = not shown because of having a very limited interpretation
2. = control predictor
3. = reference level is Mangabey
4. = z-transformed, mean and standard deviation in Table S7

Table S2B: Result of **Final Model 1.1**: Impact of dominance rank on bystander likelihood to intervene. Test parameters in bold. Estimate and Standard Error from final model after removing all non-significant higher-order interactions, X², df and p-values result of likelihood ratio test of the reduced model lacking this term with the final model. HRG = Higher-ranking Groomer, LRG = Lower-ranking Groomer, DDSI = Dynamic Dyadic Sociality Index.

| **Term** | **Estimate** | **SE** | **Χ^2^** | **df** | **p** |
| --- | --- | --- | --- | --- | --- |
| Intercept | -6.224 | 0.232 | (1) | (1) | (1) |
| Group East ^(3)^ | 0.104 | 0.296 | (1) | (1) | (1) |
| Group South ^(3)^ | 0.474 | 0.287 | (1) | (1) | (1) |
| Reproductive State Groomers (no=0, yes=1) | 0.241 | 0.148 | 2.907 | 1 | 0.088 |
| Sex HRG (female=0, male=1) | 0.042 | 0.281 | 7.200 | 1 | 0.007 |
| Sex LRG (female=0, male=1) | 0.832 | 0.257 | 10.494 | 1 | 0.001 |
| Sex Bystander (female=0, male=1) | 0.400 | 0.263 | 2.395 | 1 | 0.122 |
| DDSI HRG – Bystander ^(4)^ | 0.218 | 0.053 | (2) | (2) | (2) |
| DDSI LRG – Bystander ^(4)^ | 0.147 | 0.053 | (2) | (2) | (2) |
| DDSI Groomers ^(4)^ | -0.205 | 0.061 | (2) | (2) | (2) |
| **Rank HRG ^(4)^** | -0.051 | 0.157 | 0.037 | 1 | 0.848 |
| Rank LRG ^(4)^ | -0.195 | 0.132 | (1) | (1) | (1) |
| Rank Bystander ^(4)^ | 0.473 | 0.187 | (1) | (1) | (1) |
| **Rank Bystander * Group East** | -0.776 | 0.249 | 19.418 | 2 | 0.000 |
| **Rank Bystander * Group South** | -0.819 | 0.256 |  |  |  |
| **Rank LRG *Rank Bystander** | 0.257 | 0.113 | 5.504 | 1 | 0.019 |

1. = not shown because of having a very limited interpretation
2. = control predictor
3. = reference level is Mangabey
4. = z-transformed, mean and standard deviation in Table S7

**Table S3A: Result of Full Model 1.2: Impact of dyadic relationship values on bystander likelihood to intervene. Test parameters in bold. Estimate and Standard Error from full model, X², df and p-values result of likelihood ratio test of the reduced model lacking this term with the full model. PG = Preferred Groomer, NPG = Non-preferred Groomer, DDSI = Dynamic Dyadic Sociality Index. Full null model comparison: X² = 26.958, df = 12, p = 0.008.**

| **Term** | **Estimate** | **SE** | **Χ^2^** | **df** | **P** |
| --- | --- | --- | --- | --- | --- |
| Intercept | -6.328 | 0.277 | (1) | (1) | (1) |
| Group East ^(3)^ | -0.283 | 0.410 | (1) | (1) | (1) |
| Group South ^(3)^ | -0.031 | 0.403 | (1) | (1) | (1) |
| Reproductive State Groomers (no=0, yes=1) | 0.239 | 0.148 | 3.788 | 1 | 0.052 |
| Sex PG (female=0, male=1) | 0.401 | 0.491 | (1) | (1) | (1) |
| Sex NPG (female=0, male=1) | 0.368 | 0.486 | (1) | (1) | (1) |
| Sex Bystander (female=0, male=1) | 0.652 | 0.415 | (1) | (1) | (1) |
| Sex PG * Group East | 0.328 | 0.512 | 0.483 | 2 | 0.786 |
| Sex PG * Group South | 0.334 | 0.492 |  |  |  |
| Sex NPG * Group East | 0.350 | 0.516 | 0.525 | 2 | 0.769 |
| Sex NPG * Group South | 0.358 | 0.501 |  |  |  |
| Sex Bystander * Group East | -0.307 | 0.499 | 5.871 | 2 | 0.053 |
| Sex Bystander * Group South | -0.151 | 0.484 |  |  |  |
| Rank PG ^(4)^ | -0.201 | 0.109 | (2) | (2) | (2) |
| Rank NPG ^(4)^ | -0.235 | 0.134 | (2) | (2) | (2) |
| Rank Bystander ^(4)^ | 0.008 | 0.136 | (2) | (2) | (2) |
| DDSI PG – Bystander ^(4)^ | 0.309 | 0.183 | (1) | (1) | (1) |
| DDSI NPG – Bystander ^(4)^ | 0.155 | 0.208 | (1) | (1) | (1) |
| DDSI Groomers ^(4)^ | -0.209 | 0.151 | (1) | (1) | (1) |
| DDSI PG – Bystander * Group East | -0.240 | 0.219 | (1) | (1) | (1) |
| DDSI PG – Bystander * Group South | -0.018 | 0.214 |  |  |  |
| DDSI NPG – Bystander * Group East | 0.050 | 0.250 | (1) | (1) | (1) |
| DDSI NPG – Bystander * Group South | -0.125 | 0.244 |  |  |  |
| DDSI PG – Bystander * DDSI NPG – Bystander | 0.143 | 0.161 | (1) | (1) | (1) |
| **DDSI Groomers * Group East** | 0.027 | 0.192 | 1.299 | 2 | 0.522 |
| **DDSI Groomers * Group South** | 0.121 | 0.189 |  |  |  |
| **DDSI PG – Bystander * DDSI NPG – Bystander * Group East** | -0.134 | 0.171 | 0.582 | 2 | 0.748 |
| **DDSI PG – Bystander * DDSI NPG – Bystander * Group South** | -0.077 | 0.180 |  |  |  |

1. = not shown because very limited interpretation
2. = control predictor
3. = reference level is Mangabey
4. = z-transformed, mean and standard deviation in Table S7

**Table S3B: Result of Final Model 1.2: Impact of dyadic relationship values on bystander likelihood to intervene. Test parameters in bold. Estimate and Standard Error from final model after removing all non-significant higher-order interactions, X², df and p-values result of likelihood ratio test of the reduced model lacking this term with the final model. PG = Preferred Groomer, NPG = Non-preferred Groomer, DDSI = Dynamic Dyadic Sociality Index.**

| **Term** | **Estimate** | **SE** | **Χ^2^** | **df** | **P** |
| --- | --- | --- | --- | --- | --- |
| Intercept | -6.389 | 0.250 | (1) | (1) | (1) |
| Group East ^(3)^ | -0.293 | 0.306 | (1) | (1) | (1) |
| Group South ^(3)^ | 0.083 | 0.305 | (1) | (1) | (1) |
| Reproductive State Groomers (no=0, yes=1) | 0.261 | 0.146 | 9.206 | 1 | 0.002 |
| Sex PG (female=0, male=1) | 0.682 | 0.234 | 9.176 | 1 | 0.002 |
| Sex NPG (female=0, male=1) | 0.691 | 0.282 | 5.891 | 1 | 0.015 |
| Sex Bystander (female=0, male=1) | 0.493 | 0.292 | 2.665 | 1 | 0.122 |
| Rank PG ^(4)^ | -0.204 | 0.109 | (2) | (2) | (2) |
| Rank NPG ^(4)^ | -0.267 | 0.134 | (2) | (2) | (2) |
| Rank Bystander ^(4)^ | -0.000 | 0.135 | (2) | (2) | (2) |
| **DDSI PG – Bystander ^(4)^** | 0.216 | 0.077 | 6.228 | 1 | 0.013 |
| **DDSI NPG – Bystander ^(4)^** | 0.173 | 0.067 | 12.103 | 1 | 0.001 |
| **DDSI Groomers ^(4)^** | -0.147 | 0.071 | 10.660 | 1 | 0.001 |

1. = not shown because very limited interpretation
2. = control predictor
3. = reference level is Mangabey
4. = z-transformed, mean and standard deviation in Table S7

**Table S4: Result of Full Model 2: Factors influencing which groomer is targeted by intervening individual. Test parameters in bold. Estimate and Standard Error from full model, X², df and p-values result of likelihood ratio test of the reduced model lacking this term with the full model. Target = Groomer who was approached for grooming by intervener. DDSI = Dynamic Dyadic Sociality Index. Full null model comparison: X² = 12.682, df = 11, p = 0.382.**

| **Term** | **Estimate** | **SE** | **Χ^2^** | **df** | **P** |
| --- | --- | --- | --- | --- | --- |
| Intercept | 0.232 | 0.565 | (1) | (1) | (1) |
| Group East ^(3)^ | -0.420 | 0.604 | (1) | (1) | (1) |
| Group South ^(3)^ | -0.474 | 0.577 | (1) | (1) | (1) |
| Sex Target (female=0, male=1) | 0.038 | 0.509 | 1.286 | 1 | 0.415 |
| Sex Non-Target (female=0, male=1) | -0.061 | 0.257 | 0.514 | 1 | 0.611 |
| Sex Intervener (female=0, male=1) | 0.047 | 0.417 | 0.929 | 1 | 0.503 |
| Grooming Direction Receiver^(4)^ | 0.461 | 0.262 | 4.168 | 2 | 0.164 |
| Grooming Direction Mutual^(4)^ | 0.258 | 0.282 |  |  |  |
| Rank Target ^(5)^ | 0.335 | 0.342 | (1) | (1) | (1) |
| Rank Intervener ^(5)^ | 0.022 | 0.483 |  |  |  |
| DDSI Target – Intervener ^(5)^ | 0.381 | 0.479 | (1) | (1) | (1) |
| Rank Target * Group East | -0.529 | 0.421 |  |  |  |
| Rank Target * Group South | -0.264 | 0.412 |  |  |  |
| Rank Target * Rank Intervener | 0.010 | 0.292 |  |  |  |
| Rank Intervener * Group East | 0.010 | 0.526 |  |  |  |
| Rank Intervener * Group South | -0.084 | 0.522 |  |  |  |
| **Rank Target * Rank Intervener * Group East** | -0.295 | 0.378 | 2.040 | 2 | 0.465 |
| **Rank Target * Rank Intervener * Group South** | 0.027 | 0.367 |  |  |  |
| **DDSI Target – Intervener * Group East** | -0.596 | 0.514 | 4.150 | 2 | 0.228 |
| **DDSI Target – Intervener * Group South** | -0.173 | 0.509 |  |  |  |

1. = not shown because very limited interpretation
2. = control variable
3. = reference level is Mangabey
4. = reference level is Sender
5. = z-transformed, mean and standard deviation varied depending on selected dataset in repeated measures design

**Table S5A: Result of Full Model 3.1: Impact of dominance rank on intervention success. Test parameters in bold. Estimate and Standard Error from full model, X², df and p-values result of likelihood ratio test of the reduced model lacking this term with the full model. Target = Groomer who was approached for grooming by intervener. DDSI = Dynamic Dyadic Sociality Index. Full null model comparison: X² = 27.676, df = 15, p = 0.0237.**

| **Term** | **Estimate** | **SE** | **Χ^2^** | **df** | **p** |
| --- | --- | --- | --- | --- | --- |
| Intercept | 3.623 | 1.871 | (1) | (1) | (1) |
| Group East ^(2)^ | -3.667 | 1.883 | (1) | (1) | (1) |
| Group South ^(2)^ | -3.625 | 1.871 | (1) | (1) | (1) |
| Sex Target (female=0, male=1) | -0.152 | 0.509 | 0.039 | 1 | 0.844 |
| Sex Non-Target (female=0, male=1) | -0.141 | 0.511 | 0.040 | 1 | 0.841 |
| Sex Intervener (female=0, male=1) | 1.016 | 0.483 | 4.835 | 1 | 0.038 |
| Rank Target ^(3)^ | 1.270 | 0.690 | (1) | (1) | (1) |
| Rank Non-Target ^(3)^ | -0.196 | 1.272 | (1) | (1) | (1) |
| Rank Intervener ^(3)^ | 1.896 | 1.403 | (1) | (1) | (1) |
| Rank Target * Group East | -1.150 | 0.713 | (1) | (1) | (1) |
| Rank Target * Group South | -1.358 | 0.719 | (1) | (1) | (1) |
| Rank Non-Target * Group East | -0.077 | 1.294 | (1) | (1) | (1) |
| Rank Non-Target * Group South | 0.162 | 1.291 | (1) | (1) | (1) |
| Rank Intervener * Group East | -2.083 | 1.419 | (1) | (1) | (1) |
| Rank Intervener * Group South | -2.356 | 1.419 | (1) | (1) | (1) |
| Rank Target * Rank Non-Target | 0.032 | 0.512 | (1) | (1) | (1) |
| Rank Non-Target * Rank Intervener | 0.663 | 0.821 | (1) | (1) | (1) |
| **Rank Target * Rank Non-Target * Group East** | -0.069 | 0.562 | 0.099 | 2 | 0.952 |
| **Rank Target * Rank Non-Target * Group South** | 0.061 | 0.579 |  |  |  |
| **Rank Non-Target * Rank Intervener * Group East** | -0.866 | 0.851 | 3.658 | 2 | 0.161 |
| **Rank Non-Target * Rank Intervener * Group South** | -0.364 | 0.845 |  |  |  |

1. = not shown because very limited interpretation
2. = reference level is Mangabey
3. = z-transformed, mean and standard deviation in Table S7

**Table S5B: Result of Final Model 3.1: Impact of dominance rank on intervention success. Test parameters in bold. Estimate and Standard Error from full model, X², df and p-values result of likelihood ratio test of the reduced model lacking this term with the full model. Target = Groomer who was approached for grooming by intervener. DDSI = Dynamic Dyadic Sociality Index.**

| **Term** | **Estimate** | **SE** | **Χ^2^** | **df** | **p** |
| --- | --- | --- | --- | --- | --- |
| Intercept | 2.087 | 0.827 | (1) | (1) | (1) |
| Group East ^(2)^ | -2.340 | 0.798 | (1) | (1) | (1) |
| Group South ^(2)^ | -2.163 | 0.784 | (1) | (1) | (1) |
| Sex Target (female=0, male=1) | -0.447 | 0.464 | 0.718 | 1 | 0.348 |
| Sex Non-Target (female=0, male=1) | 0.372 | 0.451 | 0.847 | 1 | 0.391 |
| Sex Intervener (female=0, male=1) | 1.029 | 0.459 | 5.072 | 1 | 0.029 |
| Rank Target ^(3)^ | 0.269 | 0.249 | 0.943 | 1 | 0.305 |
| Rank Non-Target ^(3)^ | -0.461 | 0.238 | 3.848 | 1 | 0.051 |
| Rank Intervener ^(3)^ | 1.069 | 0.577 | (1) | (1) | (1) |
| Rank Intervener * Group East | -1.299 | 0.621 | 9.099 | 2 | 0.014 |
| Rank Intervener * Group South | -1.482 | 0.624 |  |  |  |

1. = not shown because very limited interpretation
2. = reference level is Mangabey
3. = z-transformed, mean and standard deviation in Table S7

**Table S6: Result of Full Model 3.2: Impact of relationship scores on intervention success. Test parameters in bold. Estimate and Standard Error from full model, X², df and p-values result of likelihood ratio test of the reduced model lacking this term with the full model. Target = Groomer who was approached for grooming by intervener. DDSI = Dynamic Dyadic Sociality Index. Full null model comparison: X² = 5.359, df = 9, p = 0.802.**

| **Term** | **Estimate** | **SE** | **Χ^2^** | **df** | **P** |
| --- | --- | --- | --- | --- | --- |
| Intercept | 1.416 | 0.350 | (1) | (1) | (1) |
| Group East ^(2)^ | -0.977 | 0.486 | (1) | (1) | (1) |
| Group South ^(2)^ | -1.054 | 0.472 | (1) | (1) | (1) |
| Sex Target (female=0, male=1) | -0.207 | 0.276 | 0.196 | 1 | 0.658 |
| Sex Non-Target (female=0, male=1) | -0.475 | 0.286 | 2.215 | 1 | 0.137 |
| Sex Intervener (female=0, male=1) | 0.705 | 0.273 | 6.205 | 1 | 0.013 |
| DDSI Target – Intervener ^(3)^ | 0.275 | 0.553 | (1) | (1) | (1) |
| DDSI Non-Target – Intervener ^(3)^ | -0.243 | 0.286 | (1) | (1) | (1) |
| DDSI Groomers ^(3)^ | -0.383 | 0.417 | (1) | (1) | (1) |
| **DDSI Target – Intervener * Group East** | -0.395 | 0.610 | 0.392 | 2 | 0.822 |
| **DDSI Target – Intervener * Group South** | -0.274 | 0.599 |  |  |  |
| **DDSI Non-Target – Intervener * Group East** | -0.068 | 0.523 | 2.633 | 2 | 0.268 |
| **DDSI Non-Target – Intervener * Group South** | 0.412 | 0.523 |  |  |  |
| **DDSI Groomers * Group East** | 0.460 | 0.462 | 1.149 | 2 | 0.563 |
| **DDSI Groomers * Group South** | 0.222 | 0.451 |  |  |  |

1. = not shown because very limited interpretation
2. = reference level is Mangabey
3. = z-transformed, mean and standard deviation in Table S7

**Table S7: Original means and standard deviations of z-transformed variables for all models (HRG = Higher-ranking Groomer, LRG = Lower-ranking Groomer, PG = Preferred Groomer, NPG = Non-preferred Groomer, DDSI = Dynamic Dyadic Sociality Index)**

| **Model** | **Variable** | **Mean** | **SD** |
| --- | --- | --- | --- |
| 1.1 | Rank HRG | 0.768 | 0.234 |
|  | Rank LRG | 0.515 | 0.248 |
|  | Rank Bystander | 0.614 | 0.263 |
|  | DDSI HRG – Bystander | 0.511 | 0.101 |
|  | DDSI LRG – Bystander | 0.521 | 0.097 |
|  | DDSI Groomers | 0.553 | 0.111 |
| 1.2 | Rank PG | 0.628 | 0.268 |
|  | Rank NPG | 0.654 | 0.275 |
|  | Rank Bystander | 0.614 | 0.263 |
|  | DDSI PG – Bystander | 0.564 | 0.099 |
|  | DDSI NPG – Bystander | 0.468 | 0.073 |
|  | DDSI Groomers | 0.553 | 0.111 |
| 3 | Rank Target | 0.756 | 0.249 |
|  | Rank Non-Target | 0.746 | 0.249 |
|  | Rank Intervener | 0.693 | 0.241 |
|  | DDSI Target – Bystander | 0.547 | 0.111 |
|  | DDSI Non-Target – Bystander | 0.547 | 0.120 |
|  | DDSI Groomers | 0.555 | 0.114 |
